# Diffuse gallbladder epithelial proliferation after teduglutide exposure: A case report

**DOI:** 10.1002/jpr3.70284

**Published:** 2026-10-28

**Authors:** Christopher P. Duggan, Alexandra N. Carey, Megan McGivney, Mark Puder, Antonio R. Perez‐Atayde

**Affiliations:** ^1^ Center for Advanced Intestinal Rehabilitation Boston Children's Hospital Boston Massachusetts USA; ^2^ Division of Gastroenterology, Hepatology and Nutrition Boston Children's Hospital Boston Massachusetts USA; ^3^ Department of Surgery Boston Children's Hospital Boston Massachusetts USA; ^4^ Department of Pathology Boston Children's Hospital Boston Massachusetts USA

**Keywords:** GLP‐2, intestinal failure, polyps, short bowel syndrome

## Abstract

Teduglutide, a glucagon‐like peptide 2 analog that increases mucosal proliferation in the gastrointestinal tract, was approved by the U.S. Food and Drug Administration for use in children with intestinal failure in 2019 after trials of 12‐ and 24‐week duration. We report the case of a young patient treated with teduglutide for 5 years who developed diffuse gallbladder epithelial proliferation despite persistently normal biochemical hepatobiliary markers and the absence of cholelithiasis. Long‐term use of teduglutide may warrant additional monitoring.

## 1 INTRODUCTION

Teduglutide, a glucagon‐like peptide 2 (GLP‐2) analog, has been approved for use in children with intestinal failure (IF) by the U.S. Food and Drug Administration (FDA) since 2019. Animal studies show that GLP‐2 acts to promote gastrointestinal (GI) mucosal proliferation by increasing villous height and crypt depth. Trials in children confirmed that GLP‐2 analog medication improves GI absorption of nutrients and allows reduction of and sometimes complete weaning from parenteral nutrition (PN).^1^, ^2^

Patients with IF are at risk of a number of hepatobiliary morbidities, including IF‐associated liver disease, cholelithiasis, cholecystitis, and cholangitis. Based on adult data that found that patients with IF treated with teduglutide also had hepatobiliary morbidities (in ~5% of those treated), screening for hepatobiliary disease is recommended for children prescribed teduglutide. This includes obtaining baseline serum bilirubin, alkaline phosphatase, amylase, and lipase, with repeat testing at least every 6 months.

We report a pediatric patient with IF treated with teduglutide for 5 years who developed diffuse gallbladder epithelial hyperplasia despite persistently normal biochemical hepatobiliary markers and the absence of cholelithiasis. To our knowledge, this case represents a previously unreported gallbladder finding associated with long‐term teduglutide therapy.

## 2 CASE REPORT

A 14‐year‐old female with IF was prescribed teduglutide. She had been supported on PN and fluids since infancy due to a history of jejunal atresia and bowel resection with an estimated 31 cm of residual small intestine in continuity with her distal colon. Over many years, she gradually increased the amount of enteral and oral nutrition, and PN was weaned at age 10.8 years. Intravenous fluids were continued to maintain hydration. A prior upper endoscopy and colonoscopy demonstrated normal histopathology. At the time of teduglutide initiation at age 14.4 years, she was receiving 2000 mL of intravenous normal saline nightly, 1980 calories of oral amino acid‐based formula, and 800–1000 calories from regular food. Her intravenous fluids were subsequently gradually discontinued, and her central venous catheter was removed at age 15.9 years.

Follow‐up upper GI endoscopy and colonoscopy were performed 14 months after teduglutide treatment, which revealed no colorectal polyps but identified a frond‐like, villous periampullary lesion adjacent to the ampulla of Vater, which was histologically consistent with foveolar metaplasia. Repeat upper GI endoscopy 1 year later with a side‐viewing endoscope showed similar frond‐like lesions at both the major and minor papillae; histologic examination confirmed foveolar metaplasia rather than true polyps. After discussion of the findings, she and her family elected to continue teduglutide therapy.

Serial serum bilirubin, alkaline phosphatase, gamma‐glutamyl transpeptidase, amylase, and lipase concentrations were normal. Due to her history of liver disease in infancy, she underwent serial ultrasound examinations of her liver and spleen. Six months after the initiation of teduglutide, the abdominal ultrasound was normal. Four years later (1/25), the ultrasound noted “mild gallbladder wall thickening measuring up to 2.6 mm in thickness with at least two small gallbladder polyps which are new since prior examination, both measuring up to approximately 2 mm.” She reported no abdominal pain consistent with gallbladder disease. Repeat ultrasonography 2 months later demonstrated persistent lesions measuring up to 4 mm. Due to the concern about polyp growth and medication exposure, plans for elective cholecystectomy were made, during which time she developed intermittent back pain associated with meals.

At open laparotomy, extensive adhesions were noted to the liver and gallbladder. The gallbladder was enlarged and adherent to the liver and abdominal wall. Cholecystectomy was successfully performed. Gross pathological examination demonstrated an enlarged gallbladder measuring 11 × 3.5 × 1.5 cm with a smooth tan‐pink serosal surface. Two very small polyploid lesions were noted. Histologic examination (Figure 1A–D) revealed diffuse mucosal epithelial hyperplasia with marked elongation of tortuous and villiform/papillary mucosal folds, producing prominent projections into the gallbladder lumen. Multiple sections showed glandular proliferation with intestinal and foveolar metaplasia extending into the lamina propria. A mild chronic inflammatory infiltrate was present. In addition, numerous pigmented histiocytes were present in the lamina propria. Some areas demonstrated cystically dilated glands and Rokitansky–Aschoff sinuses. No cytologic atypia or invasive growth was observed. The characteristic “cauliflower‐like” architecture typical of gallbladder polyps was absent.

**Figure 1:**
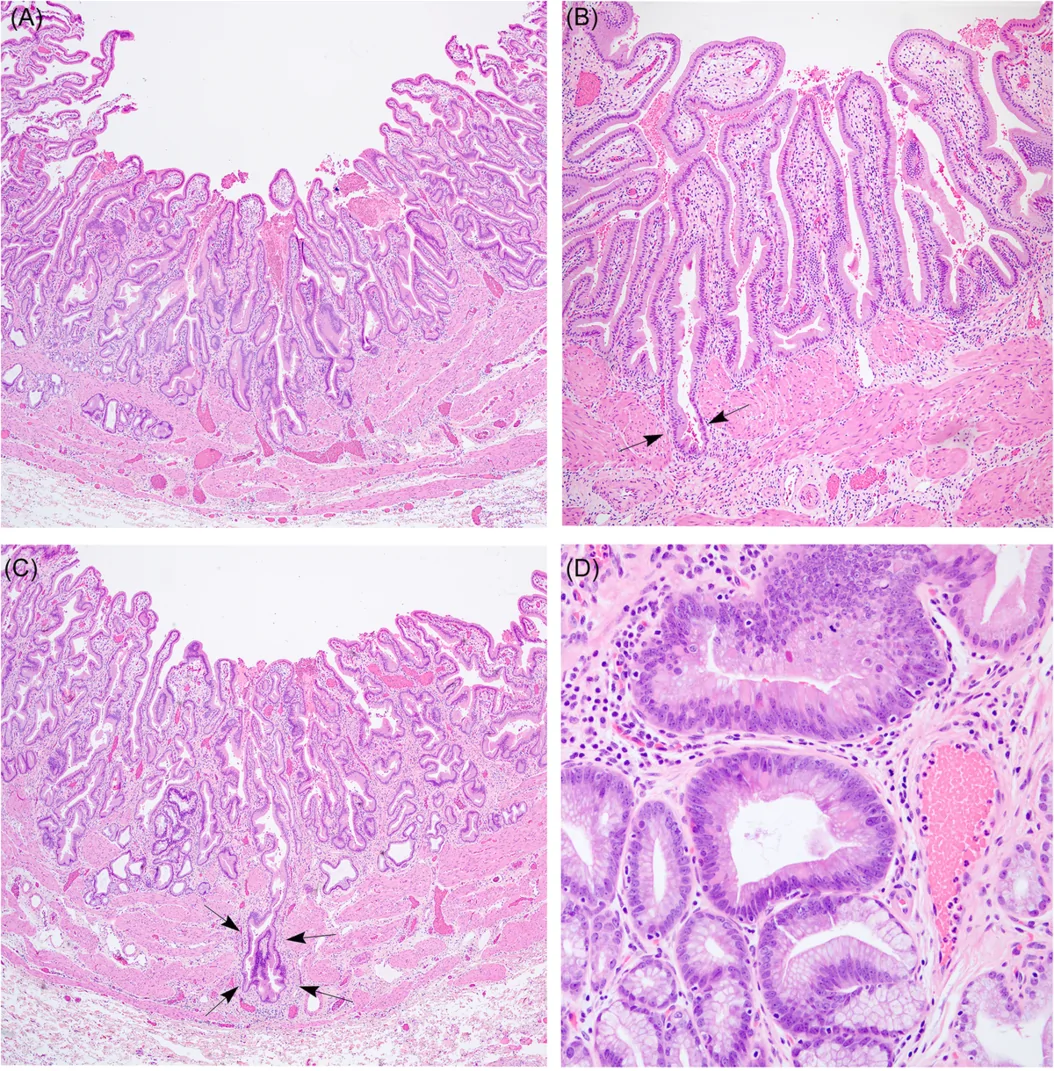
(A) Section of gallbladder wall demonstrating diffuse mucosal epithelial hyperplasia with crowded mucosal folds and architectural complexity. The mucosal surface is elongated, tortuous, and has a papillary/villiform appearance. (B) There is prominent elongation and branching of the mucosal folds, producing tall, finger‐like mucosal projections lined by biliary‐type epithelium imparting a papillary or villiform architecture. The lining epithelium appears orderly with preservation of nuclear polarity and without evidence of cytologic atypia. The lamina propria within the folds is expanded by a chronic inflammatory infiltrate. (C) Hyperplastic, chronically mildly inflamed mucosa with prominent stromal and glandular remodeling. Glands at the mucosal base appear dilated. A mucosal diverticulum or epithelial invagination herniates downward through the muscular layer, consistent with a Rokitansky–Aschoff sinus (indicated by arrows). (D) Intestinal and foveolar metaplasia characterized by numerous crowded mucinous glands and occasional Paneth cells. The glands are lined by cytologically bland cuboidal to low columnar epithelium and are associated with a chronic inflammatory infiltrate. No epithelial dysplasia or invasive features are observed.

The postoperative course was uncomplicated. The patient remains on teduglutide, maintains full oral nutrition without intravenous support, and is scheduled for repeat GI endoscopic surveillance 2 years after her most recent examination.

## 3 DISCUSSION

Limited adult data suggest that GI polyps are found in fewer than 2% of adults treated with teduglutide. We have previously noted the occurrence of gastric foveolar polyps in two children treated with this GLP‐2 medication after 1 year of therapy.^3^ Published data concerning gallbladder pathology in children treated with teduglutide are limited to a single case report of a teenager who developed acute cholecystitis 1 month after starting teduglutide in the setting of pre‐existing cholelithiasis.^4^ In that case, histopathology of the resected gallbladder included intramural hemorrhage, inflammatory cell infiltrate, multiple gallstones, and mucosal epithelial proliferation attributed to cholecystitis. In contrast, our patient had no evidence of cholelithiasis and demonstrated diffuse epithelial hyperplasia without histologic features of acute inflammation.

Studies in healthy adult males confirm that GLP‐2 inhibits gallbladder contractility,^5^ probably predisposing patients treated with GLP‐2 analogs to gallbladder diseases including cholelithiasis. While GLP‐2 receptors are present in the gallbladder, they are not clearly present in gallbladder epithelial cells but possibly exert their effects via enteric neurons.^6^ Whether GLP‐2 signaling could contribute directly or indirectly to gallbladder epithelial proliferation remains unknown.

Even before FDA approval of GLP‐2 medications for adults (2012) and children (2019) and in light of a high prevalence of symptomatic gallbladder disease, prophylactic cholecystectomy in patients with IF had been variously advocated in the literature to avoid the development of acute cholecystitis and other morbidities.^7^, ^8^ Whether this case suggests that patients with IF treated with teduglutide have an increased risk for gallbladder diseases over baseline can only be assessed by larger and longer post‐marketing surveillance cohorts.

## 4 CONCLUSION

FDA approval for teduglutide in children was based on the results of a 24‐week clinical trial^9^ and longer‐term follow‐up studies have demonstrated an acceptable safety profile.^1^, ^10^ Nevertheless, as treatment durations increasingly extend over several years, continued surveillance for uncommon or previously unrecognized adverse effects remains warranted. This case highlights a potentially novel gallbladder manifestation that clinicians should consider during long‐term monitoring of pediatric patients receiving teduglutide.

## CONFLICT OF INTEREST STATEMENT

The authors declare no conflicts of interest.

## ETHICS STATEMENT

The patient described provided written informed consent for this publication.

## Data Availability

The data that support the findings of this study are available on request from the corresponding author. The data are not publicly available due to privacy or ethical restrictions.
